# Characterization of Low- and High-Velocity Responses of Basalt–Epoxy and Basalt–Elium Composites

**DOI:** 10.3390/polym16070926

**Published:** 2024-03-28

**Authors:** Jesse Joseph Llanos, Ke Wang, Farid Taheri

**Affiliations:** Advanced Composites and Mechanics Laboratory, Department of Mechanical Engineering, Dalhousie University, Halifax, NS B3H 4R2, Canada; js205258@dal.ca (J.J.L.); ke.wang@dal.ca (K.W.)

**Keywords:** elium, basalt, fiber-reinforced composite, recyclable, environmentally friendly, numerical modelling, high-velocity impact, low-velocity impact, cost-effective analysis

## Abstract

Currently, fiber-reinforced polymer composites (FRPs) used for demanding structural applications predominantly utilize carbon, glass, and aramid fibers embedded in epoxy resin, albeit occasionally polyester and vinyl ester resins are also used. This study investigates the feasibility of employing recyclable and sustainable materials to formulate a composite suitable for load-bearing structural applications, particularly in scenarios involving low-velocity and high-velocity impacts (LVIs and HVIs, respectively). The paper presents a comparative analysis of the performance of basalt–Elium, a fully recyclable, sustainable, and environmentally friendly composite, with an epoxy-based counterpart. Moreover, an accurate and reliable numerical model has been developed and introduced through which the response of these composites can be examined efficiently and accurately under various loading states. The results of this investigation demonstrate the viability of the basalt–elium composite as a fully recyclable and sustainable material for crafting efficient and lightweight composites. Additionally, the accurately developed finite element model presented here can be used to assess the influence of several parameters on the composite, thereby optimizing it for a given situation.

## 1. Introduction

Recent years have witnessed a gradual surge in demand for fiber-reinforced plastics (FRPs). The implementation of FRPs can be observed across nearly every industry, ranging from advanced applications in aviation to the simplest components of a bicycle. The primary and foremost reasons for the utilization of FRPs are their non-corrosive and lightweight nature and high specific strength and stiffness, which often surpass those of commonly used engineering lightweight metals, such as various grades of aluminum and magnesium alloys. Lately, however, there has been an overwhelming increase in the demand for FRPs due to the relatively recently set limits for greenhouse and polluting gases and the requirements for eco-friendliness and recyclability. Additional efforts are being expended in relation to improving the ease and integration of manufacturing and reducing the overall cost of FRPs.

Commonly used FRPs consist of fibers like carbon, glass, and, in some applications, aromatic polyamide, embedded mainly in thermoset resins, such as epoxy or vinyl ester resins, and to a significantly lesser extent in thermoplastic resins. For instance, virtually all 550,000 tons of the composites consumed to make the approximately 20,000 wind turbines in the world yearly are made from glass fiber or carbon fiber–epoxy thermoset resin. 

One of the significant drawbacks of using thermoset resins is their unrecyclable nature. Plastic waste is generated during their fabrication and causes significant environmental impediments at the end of the useful life cycle of FRPs. Another notable drawback related to thermoset FRPs is their brittle nature, which makes them susceptible to damage under impact events, where, in most cases, the damage could be invisible. This can potentially compromise the structural integrity of vital structural components during impact events.

Elium©, the world’s first liquid thermoplastic resin, is fully recyclable, which, combined with its relatively high ductility and toughness, has created an opportunity to develop enhanced FRPs and address the aforementioned issues. Its recyclable nature could also potentially reduce the waste associated with thermosetting plastics. Elium’s ductility also has the potential to enhance the impact resistance of its composites, leading to longer service life and increased part durability. However, compared to the research works related to thermoset and thermoplastic composites, research on Elium composites is quite limited, especially Elium composites made with fibers other than glass and carbon, such as aramid, and especially strong and stiff eco-friendly fibers like basalt, the latter having motivated this study. Basalt fiber is considered a “green” natural fiber with properties on par with E-glass fiber. Its biodegradability and the abundant availability of its base material (igneous rocks) render it a promising fiber for structural applications. This fiber has been considered an excellent natural fiber due to its mechanical properties, especially under elevated temperatures, and its tribological properties render it a great candidate for replacing currently used synthetic fibers in applications from the automotive industry to aerospace. However, there is a lack of studies considering its response within different resins and the resulting composites’ behaviors under various mechanical and thermal loadings, as well as their wear and fatigue responses. Therefore, a significant amount of research is required to enrich the current database and make basalt fiber and the recyclable thermoplastic Elium resin the composite material of choice for various load-bearing applications. Of course, there are currently other issues that impede the wider use of basalt fiber, such as its current cost and its sizing (from a chemical perspective) and hence its compatibility with various resins, in addition to its general availability in various forms, compared to E-glass and carbon fibers. Moreover, the current studies that have investigated Elium composites have mostly considered glass and carbon composites and low-velocity impact critical loading statuses. Therefore, it is believed that there is a clear need for a systematic investigation to establish the performance of these composites under low- and high-velocity impact scenarios. 

In summary, the work presented here experimentally characterizes the low-velocity impact (LVI) and high-velocity impact (HVI) responses of various composites fabricated using the novel Elium 150 thermoplastic and epoxy resins with basalt fabric. Furthermore, a numerical model is developed by which the low- and high-velocity impact responses of Basalt-Elium are accurately simulated. This model can be used to ascertain several parameters that would otherwise require exhaustive and costly experimental evaluations.

## 2. Literature Review

This section outlines the fundamental scientific foundation and relevant research investigating the low- and high-velocity impact characterization of Elium-based composites, noting that most readers are familiar with the basic mechanical testing of FRPs.

### 2.1. Low-Velocity Impact (LVI)

Since two of the most prominent and positive attributes of Elium compared to brittle thermoset resins are its ductility and toughness, this resin is expected to perform superiorly under collision and impact events. As also stated, the laminated nature of FRPs and the susceptibility of FRPs to delamination, especially invisible delamination, renders the necessity of considering impact events in designs that utilize FRPs. Consider a material like aluminum under an impact loading condition. An impact into aluminum will potentially create a large deformation or, in most critical situations, a penetration. In the case of most metallic alloys, such outcomes would potentially be non-detrimental to the overall integrity of the impacted structural components due to the ductility of most metals and their plastic deformation. Most metals also offer a strain-hardening effect, thereby offering an additional degree of sustenance in relation to such events, an attribute not shared with most laminated FRP composites.

In an impact event, since most FRPs lack any through-thickness reinforcement, such loading develops localized interlaminar shear and normal stresses, in turn causing damage to the composite. These stresses are usually the initial causes of structural failure on the microscopic level. For this reason, design failure strains are usually as low as 0.5% [1,2]. This low design failure strain renders an FRP with a significantly underutilized in-plane material strength. In general, impact is categorized based on impact velocity into low-, high-, and hyper-velocity categories. At low velocities, the interaction between the material and the impactor is relatively long enough for the effect of the event to extend beyond the zone of impact. Cantwell and Morton classified an LVI as an impact event that takes place below 10 m/s [2]. This value gives a good approximation for most materials, but Davies and Robinson [3] outlined a more accurate method by considering the through-thickness stress wave and its effects, using the following equation:

(1) $$\varepsilon =\frac{V_{o}}{C}$$

where ε is the strain in the material, $V_{o}$ is the impact velocity, and C is the speed of sound in the material. The above equation indicates that the stress wave’s dominant starts taking place between 10 and 20 m/s [3].

The failure modes observed as a result of an impact event are categorized into three groups: delamination, matrix cracking, and penetration. The delamination mode occurs when a strong difference gradient exists in the bending stiffness between fiber layups [4]. Such delaminations have an oblong appearance, with the longer axis being parallel to the fiber direction [4]. Dorey [5] stated that the development of delamination would be more likely in composites with a shorter length. This observation, combined with that of Liu [4], is believed to reveal the most critical combination in causing delamination. The matrix cracking mode occurs when the absorbed energy leads to the formation of micro- and macrocracks. Unlike delamination, matrix cracking occurs across fibers. This effect usually occurs when the impact energy is within a smaller range of values, usually below 5 J. It should be noted that damage to the matrix is usually the first form of damage during an impact event and is usually located in parallel planes to the fiber direction, caused by the existence of a differential gradient between the properties of the fiber and matrix [6]. These cracks are commonly categorized into two categories: (a) bending cracks and (b) shear cracks [7]. Bending cracks are usually formed perpendicular to the fibers and originate between fiber layers at the boundary of the fiber–matrix interface, whereas shear cracks, which are usually oriented at a 45° angle to the fibers, are formed as a result of large traverse shear stress often due to an impact [7]. The penetration mode occurs under high-impact energy levels, resulting in significant damage to both the matrix and the fibers. The significant damage to the matrix forces the fibers to sustain the load. Cantwell and Morton [8] found that the mode of penetration that has the highest energy absorption is shear-out failure. This failure mode is recognized by a plug of material sheared out of the composite panel, absorbing between 50 and 60% of the impact energy, depending on the thickness of the composite [2,8].

In general, since most load-bearing FRPs are made of thermoset resins (primarily, epoxies), which are generally brittle and can sustain relatively low failure strain, the full fiber strength is underutilized, reducing the feasibility of composites in many applications. It is worth mentioning that Sela and Ishai [9] found that improvement in the fracture toughness of resin can lead to failure strain values 50% higher than those of the commonly used epoxies. They also demonstrated that the use of thermoplastic resins like poly-ether-ether-ketone (PEEK) improved the fracture toughness of their composite by an order of magnitude, though it resulted in poor fiber–resin interface bonding [9].

### 2.2. High-Velocity Impact (HVI)

**Figure 1:**
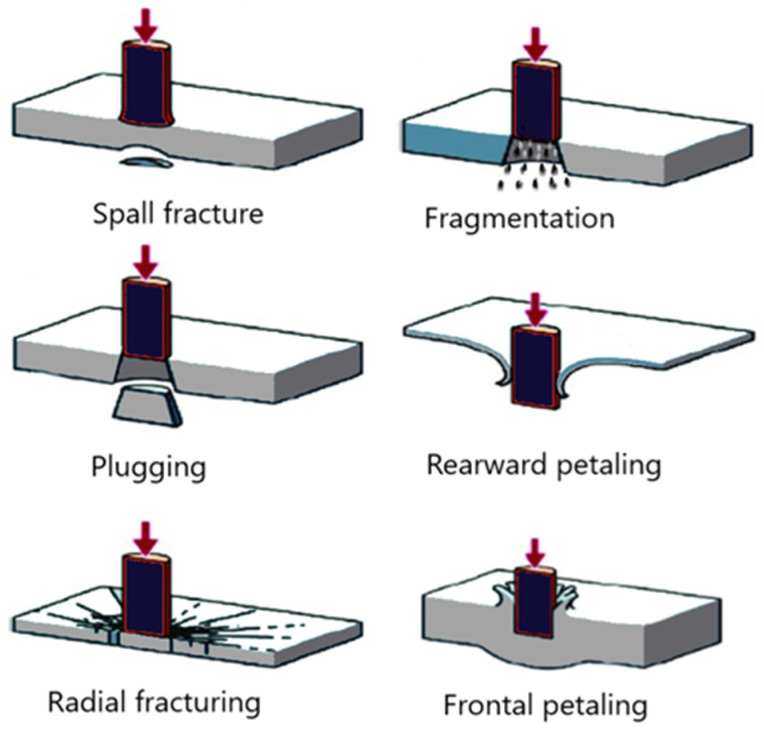
Typical HVI damage modes.

HVI plays a critical role in engineering designs involving high speeds (e.g., aviation, automotive, space, and speedboat industries). In an HVI event, the stress wave effects dominate the event. The velocity domain in which stress wave effects become dominant can be established by using the simple equation introduced earlier (1). Olsson found that during an impact event, a continuum of three wave types exists [10]. Three-dimensional dilatational waves dominate impact events where the event duration approaches the through-thickness wave propagation period [10,11]. As the time of the impact event increases, the waves transition to flexural waves until a quasi-static state is achieved [10,11]. Olson also discovered that the quasi-static waves were affected by the size of the target and the boundary conditions, but the parameters had no influence on the dilatational, flexural, and shear waves [12]. It should be noted that the failure modes described earlier for LVI events also hold for HVI events. The major difference between the two is that in an LVI event, one mode usually dominates the failure mode, whereas in HVI events, a mixture of the modes is generally experienced (see Figure 1).

Another parameter that significantly affects the ability of composites to absorb energy in HVIs is the angle at which the impactor strikes the target; the larger the angle, the more adverse the outcome [13]. Siva Kumar and Bhat concluded that small increases in the angle of impact had a minimal effect on the energy absorbed; however, once the angle of incident surpassed 30°, an increase in the absorbed energy would be observed [13]. The penetration modes have a further delineation depending on the material and its properties. During an impact event, a compression–tension alternating wave is also generated (especially in low-density materials). If this wave exceeds the tensile strength of the material, radially spaced cracks will occur, which is common in materials with a compression strength greater than the tensile strength [14,15].

Another well-known failure mode is referred to as “pedaling”, which occurs when a high amount of tensile stress is developed at the backside of an impacted FRP panel during an event and released once the damage has occurred. Another phenomenon known as “fragmentation” is caused by localized pulverization of the material upon impact and is seen more prominently in brittle materials [14,15]. Finally, “plugging” is where a cylindrical portion of the impacted material is ejected during an impact event, which is caused by a high amount of through-thickness shear stress development around the borders of a blunt projectile [14,15]. The damage modes are presented graphically in Figure 1.

An important metric in HVI is the “ballistic limit”, which represents the velocity at which a material is penetrated. This value varies for each material and configuration. Extensive work has been undertaken to develop predictive models for both composites and common engineering materials [15,16]. These models have been shown to produce somewhat reasonable approximations; however, the accuracy is not adequate for engineering applications [16,17]. 

Ferriter et al. [17] investigated the effectiveness of the following models for establishing ballistic limits; the models considered were the bisection method, the Jonas–Lambert method, the Vs and Vr relationship method, the Golden Ratio method, the residual energy vs. the angle of the projectile relationship method, and the residual energy vs. Vr relationship method. They concluded that the bisection method gave the lowest error values of 1 m/s with a sample size of 5.5 specimens [17]. The projectile geometry has a significant influence on the modes of failure in HVIs [18]. Mines et al. [18] found that an impactor with a hemispherical geometry had the highest target energy absorption for stitched fabric. In contrast, a flat impactor had the highest energy absorption for woven fabric. They concluded that flat impactors would cause material failure mainly due to punching shear, while the round and conical impactors would cause a mixed failure mode involving tensile, shear, and bending stresses, whereas conical impactors would also generate a mixed failure mode of tensile and shear failure modes.

### 2.3. Elium Resin

Elium resin is an epoxy-like low-viscosity liquid thermoplastic resin with the chemical name poly methyl methacrylate (PMMA), first introduced in 2009 by Arkema S.A. (France) as a potential replacement for epoxy resin. Elium resin undergoes the chemical crosslinking process by radical polymerization, where its monomer methyl methacrylate (MMA) transitions to its polymer form, PMMA, through the use of a peroxide catalyst [19,20]. Incorporating a thermoplastic in fabricating FRPs leads to a significant improvement in the through-thickness performance [21], which is governed [19,20] by the fracture toughness of resin and fibers. Bhudolia et al. [21] demonstrated the effect of Elium on the fracture toughness of composites fabricated using three different fibers. The results of their study showed that the addition of Elium increased the mode II fracture toughness of composites constructed with ultra-high-molecular-weight polyethylene fibers by 33%. Barbosa et al. [19] conducted similar research and examined the influence of Elium on the fracture toughness of composites made with carbon fiber, which resulted in significantly (40%) higher fracture toughness compared to a comparable carbon–epoxy composite.

In general, several studies have shown that the mechanical properties of Elium-based composites are quite similar to those of common thermoset resin composites [20,22,23]; however, the properties susceptible to resin–fiber interface strength are somewhat affected by the relatively poor resin–fiber interface strength of Elium compared to its thermoset counterparts. For instance, Yaghoobi and Taheri [22] found that a basalt–Elium-based composite had a 5% lower stiffness but a 23.5% higher tensile strength when compared to an equivalent epoxy–basalt composite. Once these values were normalized, the 5% difference in modulus was mostly removed, while a tensile strength increase was realized at 28.4% [22]. Bhudolia et al. [23] found a similar flexural response between Elium and epoxy–carbon FRPs. They demonstrated that the annealing of Elium, which increased the polymerized resin sites, resulted in a 21% higher flexural strength and an 11% higher modulus than the laminate with no annealing [23]. 

As stated earlier, thermoplastics have the advantage of being recyclable, though recycling comes with a few potential drawbacks, such as void formation, impurity inclusion, and weakening properties in the crystal structure. However, one can reduce such drawbacks significantly by incorporating effective quality control. The recyclability of flax–Elium composites was investigated by Allagui et al. [24]. A decrease in the tensile properties of the composites was found after recycling. Their further analysis revealed that the change in the elastic properties was mainly due to the reduction in the fiber size, which resulted from the incorporated recycling approach. Allagui et al.’s investigation was further expanded by Sahki [25], who investigated the influence of recycling methods on E-glass–Elium and basalt–Elium composites. His test results showed minimal differences in the properties between glass and basalt composites. It also achieved complete recycling of the resin with minimal loss in the properties of the recycled composite by using solvolysis/dissolution [25]. 

It should be noted that recycling, facilitated by the weakened Van Der Waals interactions resulting from heating/melting thermoplastic resins, also enables cured resin to be welded to itself. Bhudolia et al. [26] investigated the fatigue response of ultrasonically welded carbon–Elium composites by comparing them to adhesively bonded joints. They observed an increase in the fatigue strength in the range of 7–12%. Elium composites also offer improved vibration-damping characteristics compared to epoxy-based composites. This finding was verified by another study by Bhudolia et al. [27], who observed a 27% increase in the structural damping of Elium-based composites compared to epoxy-based composites.

The impact characteristics of Elium composites have also been investigated by researchers; however, the volume of research concerning LVIs of Elium composites is relatively limited, especially when one considers HVI-related studies. Bhudolia and Joshi [28] investigated the low-velocity impact response of a carbon–Elium composite against a carbon–epoxy composite. The Elium-based composite was observed to undergo elastic deformation 53% higher than that of its epoxy counterpart. Moreover, the authors observed that comparatively (58%) more absorbed energy was absorbed by the Elium-based composite before the onset of a major failure, which was facilitated by a significant elastic–plastic deformation response [28]. Kazemi and his co-workers have conducted several studies investigating the LVI responses of Elium-based composites. In one of their studies [29], they observed a remarkable increase of 240% in the structural integrity of their post-impact specimens of an Elium–carbon composite compared to a carbon–epoxy composite. Other researchers have also reported several similar investigations [28,29,30,31,32,33,34]. However, as mentioned previously, there is a clear lack of HVI-related investigations on Elium-based composites compared to commonly used epoxy-based composites. One such notable study is by Libura et al. [35], who investigated the effect of fatigue and aging on the ballistic limit of glass-woven reinforced Elium laminates. They found that fatigue and aging deteriorated the fiber–matrix interface, thus leading to a reduction in the stiffness and ballistic performance of the composite.

It should be noted that there are other recyclable and biobased epoxy-based resins, such as Recyclamine^®^ biobased epoxy resin, which has been claimed to provide 100% recyclability. However, the recyclability of such resins must be facilitated by the use of special chemicals [1]. Moreover, to the best of the authors’ knowledge, there have been no studies investigating the impact performance of such biodegradable epoxies.

### 2.4. Reinforcing Fibers

Efforts to promote the use of sustainable fibers are primarily devoted to natural plant-based fibers. However, recently, basalt has attracted considerable attention. From the perspective of non-plant-based mineral natural fibers, basalt and wollastonite are the most used mineral-based natural fibers [36]. One of the primary reasons for the recent attention to this fiber is its low energy needs, low carbon footprint, and excellent mechanical properties that are less susceptible to temperature. Moreover, they exhibit good chemical stability. The tensile strength of basalt fibers is on par with those of E-glass and some carbon fibers and superior to that of aramid fiber, though its modulus is very similar to that of E-glass fiber, which places it below aramid and carbon fibers [2]. Various attributes of basalt fiber, such as its performance alone or in conjunction with natural fibers such as hemp and flax, are thoroughly surveyed in a review article by Dubey et al. [37].

As stated, due to the stable mechanical properties and the good corrosion resistance of basalt, this fiber is used in several electrical applications, such as in new conductors, insulated pull rods, and composite cross-arms and designability. The mechanical properties of basalt fiber and its composites are reported in Table 1. The basalt fabric used in this research is a continuous fiber stitched mat biaxial (0/90) fabric with a fiber weight of 516 g/m^2^. The mechanical properties of the basalt fiber were obtained through the producer’s website [34], whereas the tensile and shear properties, void contents, and fiber volumes of the composites were evaluated in-house following ASTM D3039, ASTM D3518, and ASTM D2734-16, [38,39,40] respectively.

At this juncture, it is hoped that the potential applications of basalt as an effective and environmentally friendly reinforcing fiber can be appreciated. As stated earlier, this mineral-based fiber offers remarkable properties on par with those of E-glass fiber, and its other positive attributes have been briefly noted above. It is believed that one could obtain an effective, fully recyclable composite by combining this fiber with the thermoplastic Elium resin that would be suitable for various load-bearing applications, especially those required in harsh environments. It appears that this fully recyclable and sustainable composite has great potential for use in various applications. However, a richer and more extensive database is required for this composite to reach the fruition it deserves.

### 2.5. Motivation and Objectives

The main objectives of the work presented here were to examine the viability of the basalt–Elium composite as a viable lightweight composite material in applications that are subjected to impact loading. Therefore, first, the low- and high-velocity impact responses of basalt–Elium were characterized and compared to the responses of basalt–epoxy. Secondly, a robust and fairly accurate numerical model was developed for predicting the impact performances of such composites under more complex high-velocity impacts. The aim of the model was also to establish the “limit velocity” of the composite. Limit velocity is an important parameter to be considered when designing a structural system subjected to high-velocity impact loading, requiring extensive resources and personal time, and hence it involves a costly task. This model can also be effectively used to optimize the performance of composites when formed in complex geometries and under various combined loading scenarios.

## 3. Materials and Specimen Preparation

### 3.1. Fabrication of the Composites

**Figure 2:**
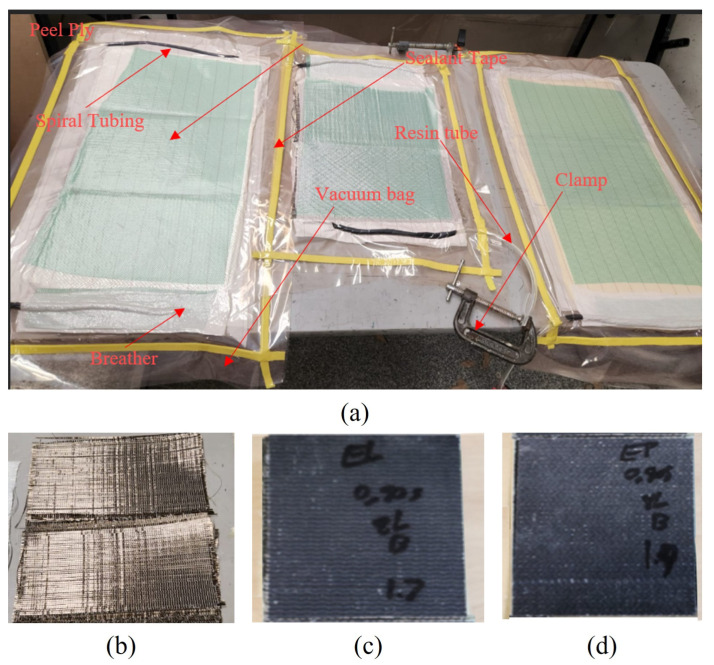
(**a**) Vacuum resin infusion setup. (**b**) Biaxial basalt fabric laid before resin infusion. (**c**) Typical Elium–basalt specimen. (**d**) Typical Elium–epoxy specimen.

The FRPs used in this study were fabricated in panel form with a fiber orientation of [0/90]_2,s_ using vacuum-assisted resin infusion molding (VARTM). Compared to vacuum-bagging hand-layup methods, the VARTM procedure provides more consistent part quality. Moreover, it facilitates the low-oxygen environment Elium requires. Finally, the fiber–resin ratio is more controllable. The FRP panels were fabricated using a resin-to-fiber weight ratio of 1:1. The vacuum-bag assemblies were left to cure under vacuum conditions for 24 h. The various reinforcing fabrics and the vacuum-bagged systems are shown in Figure 2. The FRPs were fabricated using epoxy and Elium resin. The layup sequence of each FRP was [0,90]_2,s_. 

### 3.2. Specimen Preparation

The low- and high-velocity impact specimens were manufactured using the same techniques as described in Section 3.1 with the selected fiber orientation of [0/90]_2,s_. The specimens, with dimensions of 150 × 100 mm^2^ as per the ASTM 7136 [42] guidelines, were extracted from the panels using a wet saw equipped with a diamond-coated blade. The specimen thickness was established using Equation (2), which was developed by Reid and Zhou [16].

(2) $$v_{b}=\frac{\pi \Gamma \sqrt{\rho_{c}\sigma_{e}}D^{2}t}{2m}\left[1+\sqrt{1+\frac{8m}{\pi \Gamma^{2}\rho_{c}D^{2}t}}\right]$$

where $v_{b}$ is the ballistic limit, t is the panel thickness, $\sigma_{e}$ is the static linear elastic compression limit, ρ is the density of the composite panel, Γ is the projectile constant, D is the projectile diameter, and m is the mass of the projectile. For an estimation of the necessary panel thickness, the values of the terms related to mechanical properties were taken from the experimental results and can be seen in Table 2. The calculated thickness ensured that specimen penetration would take place within the velocity range capabilities of the gas gun.

## 4. Experimental Investigation

### 4.1. Low-Velocity Impact Test

#### 4.1.1. Test Setup and Procedure

**Figure 3:**
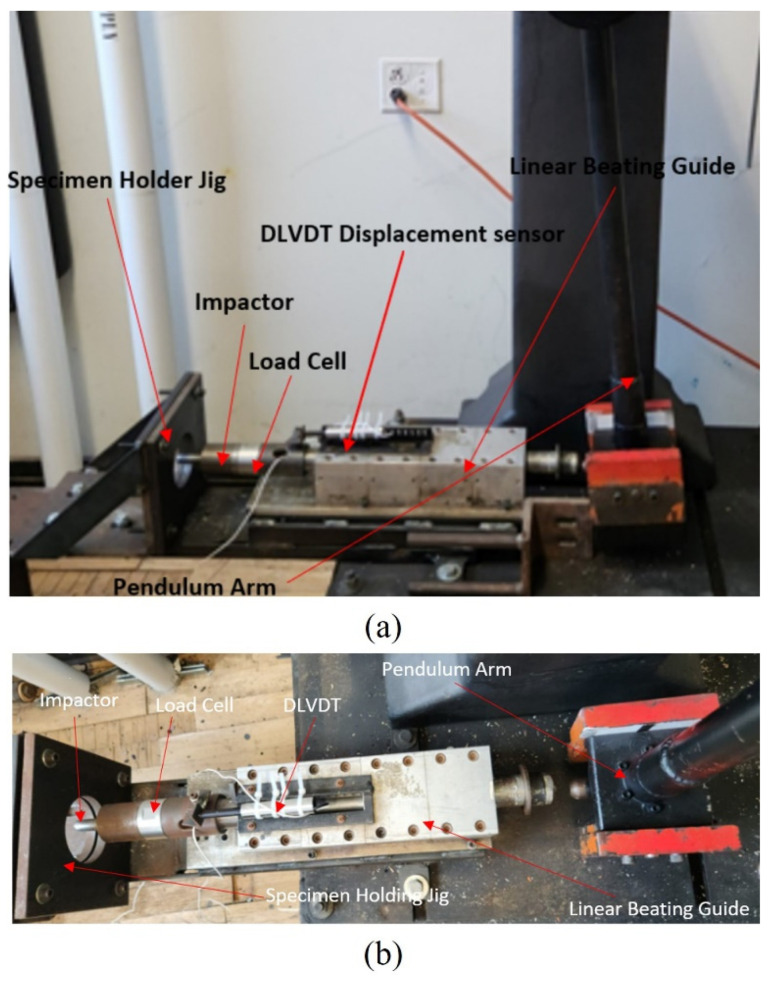
Low-velocity impact test setup: (**a**) front view; (**b**) top view.

The experimental setup for the LVI test implemented a modified Charpy impactor (see Figure 3). The impactor arm is manually raised to the desired angle, which is pre-calibrated to the required energy level. Once the arm is at the desired angle, the brake lever at the pivot is engaged, holding the arm in place. Once the brake is released, the arm swings forward, impacting the impactor contact point on the impactor propulsion guide equipped with a series of roller bearings. It is positioned so that the linear direction extending from the arc of the impactor at the point of contact forms a tangent line.

The impactor is cylindrically shaped with a hemispherical tip and has a mass of 5.822 kg. A Dytran 1060 dynamic load cell with a 225 kN capacity was placed before the impactor to record the compression force response generated by the impactor. A dynamic linear variable differential transformer (DLVDT) was used to measure the deformation of the specimen’s center, thus measuring the central displacement and indentation depth of the specimen. The signals from the DLVDT and the load cell were collected via a Compact DAQ NI Chassis cDAQ-9172 (National Instruments, San Jose, CA, USA), which was connected to a laptop, and the data were processed using LabVIEW 2015 software. The specimens were sandwiched and restrained by two rigid steel plates. The clamped plates had a square shape with a circular opening with a diameter of 80 mm instead of a square opening, providing axisymmetric restraint for the specimen, thus ensuring an even stress distribution at the boundaries of the specimen. The assembly was bolted rigidly to the main rigid platform, as shown in the above figures.

To ensure that each specimen was restrained uniformly and asymmetrically, the specimen holding jig was made to have a circular opening with a diameter of 85 mm instead of a square opening. A circular opening facilitates even restraint, thus ensuring even stress distribution at the boundaries by the holder. The LVI testing equipment was calibrated precisely with the details provided in reference [43]. Each FRP-type specimen was subjected to three impact energy levels (i.e., 25, 40, and 55 Joules), and three specimens were tested at each energy level.

#### 4.1.2. LVI Test Results

**Figure 4:**
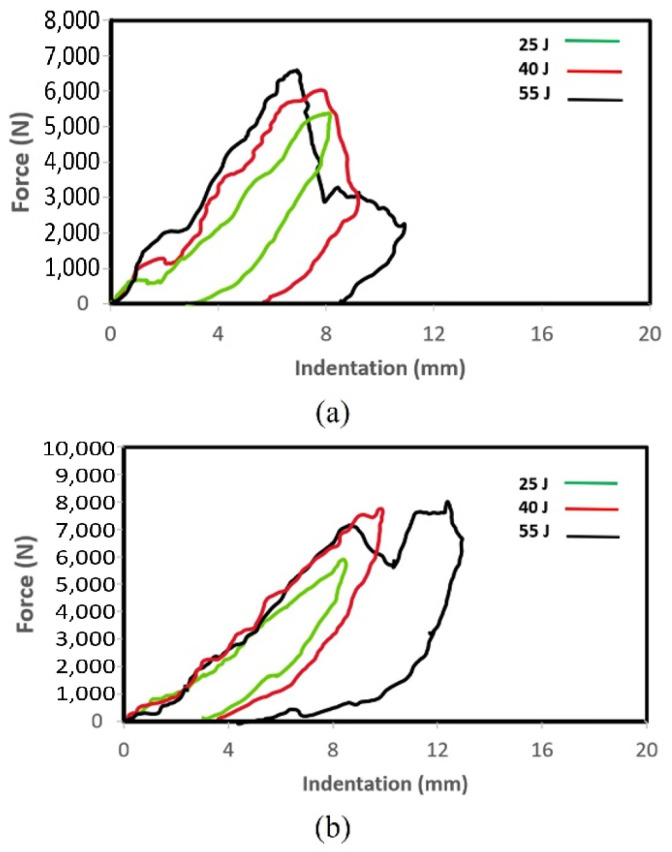
Plots of LVI impact force vs. indentation depth for (**a**) basalt–epoxy and (**b**) basalt–Elium.

**Figure 5:**
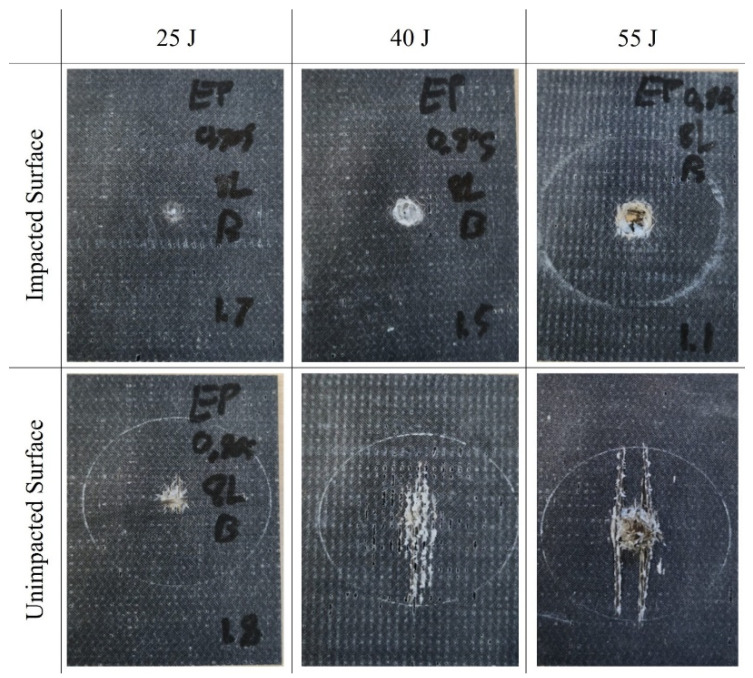
Typical post-LVI views of basalt–epoxy specimens.

**Figure 6:**
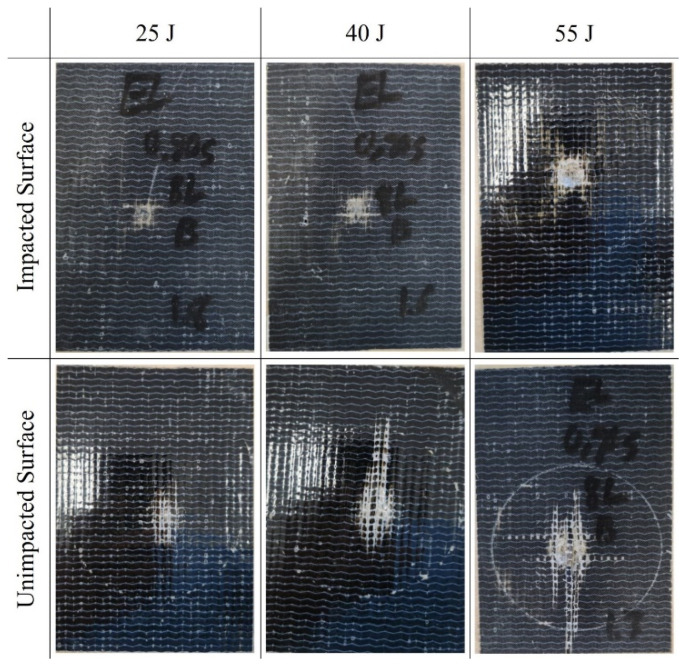
Typical post-LVI views of basalt–Elium specimens.

The load–displacement curves for the two types of composites tested under low-velocity impacts are presented in Figure 4. The average load-bearing capacity of the two composites subjected to various impact energies is summarized in Table 3. The superior load-bearing capacity of basalt–Elium was immediately apparent. Moreover, the relatively lower coefficient of variation signified more consistency in the results, which were attributed to a more ductile response of basalt–Elium compared to basalt–epoxy. Moreover, the typical post-impact characteristics of the two composites at various impact energies are illustrated in Figure 5 and Figure 6.

As can be seen in Figure 4, at 55 Joules, the low-velocity response of both basalt–epoxy and basalt–Elium shows a spike in the force during the impact, which is short-lasting (0.004 s). The short duration is due to the penetration experienced by some plies, after which the load capacity is decreased and then resisted by the remaining plies, as can also be seen in Figure 5 and Figure 6.

At the lowest impact level, both composites endured insignificant damage with almost similar impact capacities; however, basalt–Elium exhibited a significantly higher impact energy absorption capacity compared to basalt–epoxy, which is attributed to the more ductile and tougher nature of the resin.

### 4.2. High-Velocity Impact 

#### 4.2.1. Test Setup and Procedure

**Figure 7:**
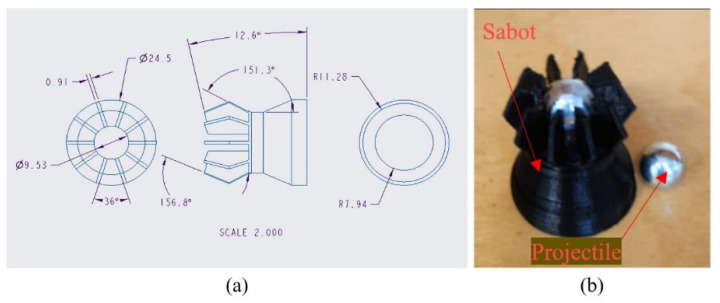
(**a**) Specifics of the designed sabot. (**b**) A 3D-printed sabot hosting a projectile.

The HVI tests were conducted using a gas gun operated with compressed air, designed and fabricated in-house. A 9.53 mm diameter solid stainless-steel ball was used as the projectile, which was selected based on the reasoning provided by Reid and Zhou [16]. The breach located at the rear of the 25 mm diameter barrel of the gun accommodates the projectile. The projectile is hosted by a sabot (see Figure 7) to facilitate the efficient use of the propellant by maintaining a proper seal with the barrel’s internal wall, which is propelled by an automated system controlled by an Arduino digital microcontroller. An electromechanical solenoid valve that is closed when uncharged but opens once charged propels the projectile. Once the sabot is ejected from the barrel, it is arrested by a sabot arresting system, which frees the steel ball to propel toward the target.

**Figure 8:**
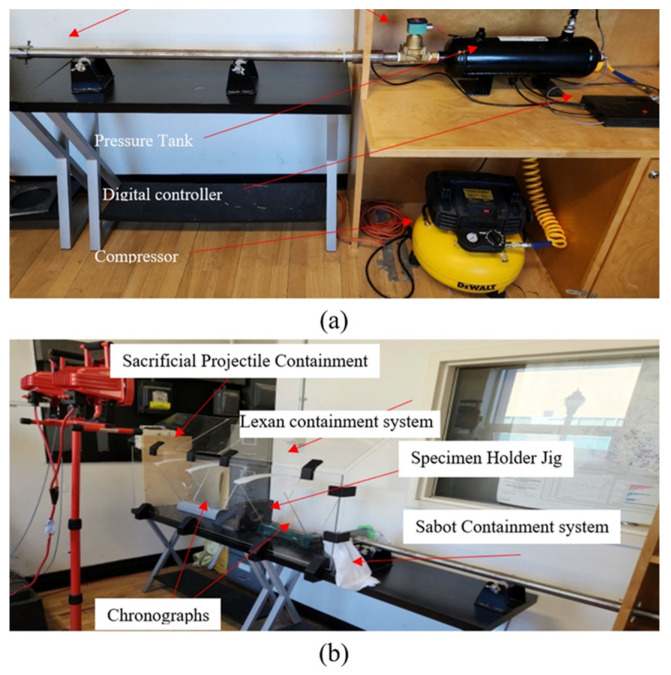
High-velocity impact test setup: (**a**,**b**) right and left sections of the setup, respectively.

As stated, the gas gun used compressed air released from a pressurizing system consisting of a pressure tank with a manufacturer-rated maximum pressure of 1.38 MPa (200 psi) and an external pressure reservoir with an electrically powered air pump (see Figure 8). Even though the pressure tank has a manufacturer-rated maximum pressure of 1.379 MPa, it was fitted with a safety release valve programmed to actuate at 1.03 MPa (150 psi). The gas gun has a velocity range of 85–190 m/s. A 12 mm thick protective Lexan shields the system. The sacrificial backing panel was a 60 mm layer of plywood, which provided a good stopping capability and prevented the potential ricochet of the projectile. 

The HVI specimens had the same dimensions as the LVI specimens (i.e., 100 mm × 150 mm), as recommended by ASTM D7136 [42]. The same specimen-holding plates used to restrain the LVI specimens were also used to support the specimens tested under HVI. Considerable effort was expended to optimize the sabot and calibrate the gas gun, which, due to limited space, will not be discussed here.

The projectile’s velocity was monitored by two ballistic chronographs and digital and analogue pressure gauges. The two precision chronographs, fabricated by Caldwell and Competition Electronics, were placed before and after the test specimen, enabling the monitoring of the velocities and energies of the projectile before and after an impact event. They are capable of measuring velocities between 1 and 3000 m/s with a manufacturer-rated accuracy of ±0.25% and ±0.5%, respectively, which is achieved through the implementation of 48 MHz processors [44,45]. 

The HVI tests were performed using a 9.53 mm dia. steel spherical projectile with a mass of 3.5 g. Six specimens were used per composite type. The velocity before and after were recorded using the two chronographs.

#### 4.2.2. Test Procedure

The HVI tests were performed in accordance with the US Army Research Laboratory’s v_50_ method [17]. This method is based on the principle that a function exists that describes the behavior of a projectile after impact and penetration. According to the document, this method can accurately obtain the ballistic limit with a tolerance of 1 m/s with an average of 5.5 impact events [17]. 

This function is continuous and differentiable; therefore, a point exists on that function which is the ballistic limit [17]. First, a range velocity was selected, which included the assumed limit velocity. For this experiment, the entire velocity range of the gas gun was selected due to the limited projectile velocity generated by the gas gun. Once the gas was triggered and the sabot was propelled, the velocity of the projectile was picked up by the chronographs and recorded. 

The measured initial and residual velocities can be fitted using Recht and Ipson’s module [46], which can be expressed as Equation (3).

(3) Vr=aVip−Vlp1p

where $V_{r}$ is the residual velocity, $V_{i}$ is the initial velocity, $V_{l}$ is the ballistic limit velocity, and a and p are characterizing constants for each projectile and target combination. If the projectile is rigid, the value of p=2 can be applied [47].

#### 4.2.3. High-Velocity Impact Test Results

The pre- and post-impact velocities were captured using the chronometers and then processed to obtain the ballistic limits. The ballistic limits and Recht and Ipson’s parameters are reported in Table 4. 

**Figure 9:**
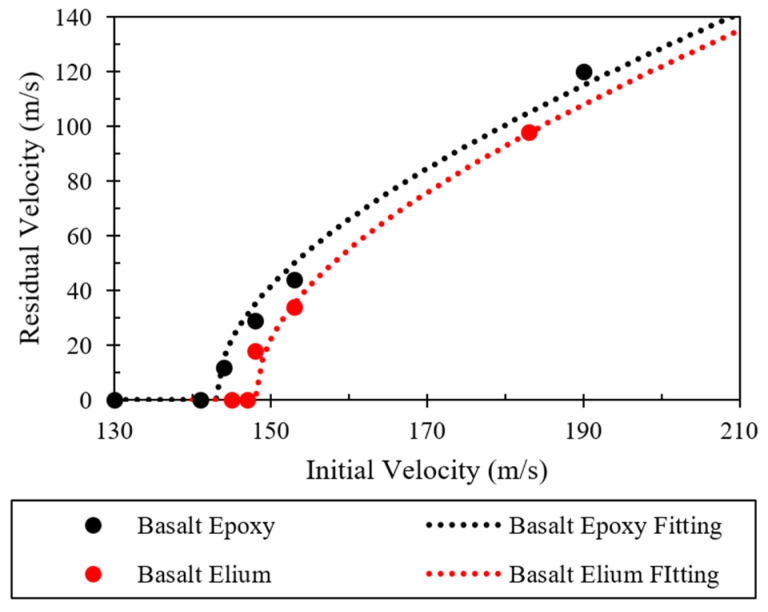
HVI results fitted using Recht and Ipson’s model.

The residual velocity vs. the initial velocity for both materials and the fitted Recht and Ipson’s curves are plotted in Figure 9. As can be seen, basalt–Elium consistently absorbed more energy than basalt–epoxy.

**Figure 10:**
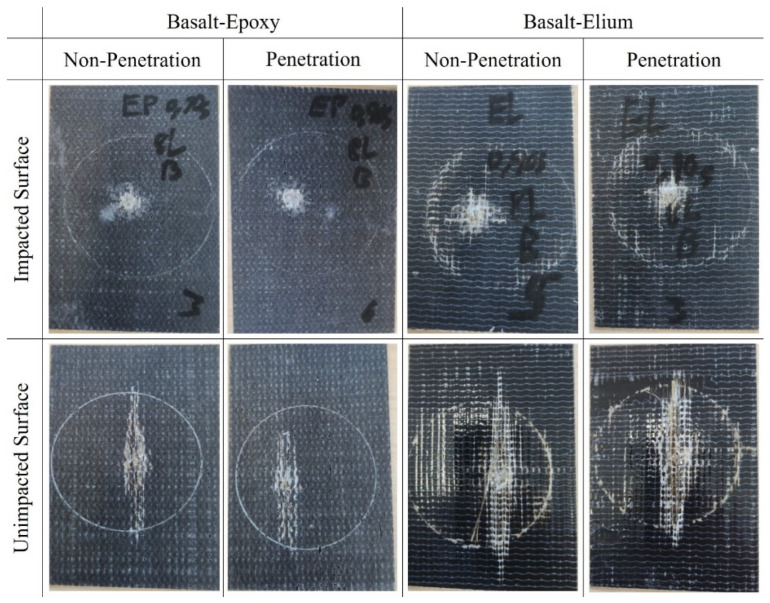
Typical post-high-velocity impact damage patterns on the surfaces of typical basalt–epoxy and basalt–Elium specimens.

The post-impact damage patterns for the two types of composites are illustrated in Figure 10. The failure modes for basalt–epoxy can be characterized as fiber pull-out, fiber tear-out, and delamination, with the most dominant failure mode being fiber pull-out. The basalt–Elium composite also exhibited fiber tear-out, fiber pull-out, and delamination with apparently larger damage regions since it endured more energy absorption. The observation of local deformation in basalt–Elium is a function of the inherent characteristic of thermoplastic composites. In other words, despite the similarities of the failure modes of the two composites, the characteristics of these modes differ for the two plastics. Specifically, the failure regions had sharper borders in the basalt–epoxy specimens due to the brittleness of epoxy, as the projectile protruded through the composites, whereas the basalt–Elium specimens exhibited a rounded and more flexible damaged region. 

**Figure 11:**
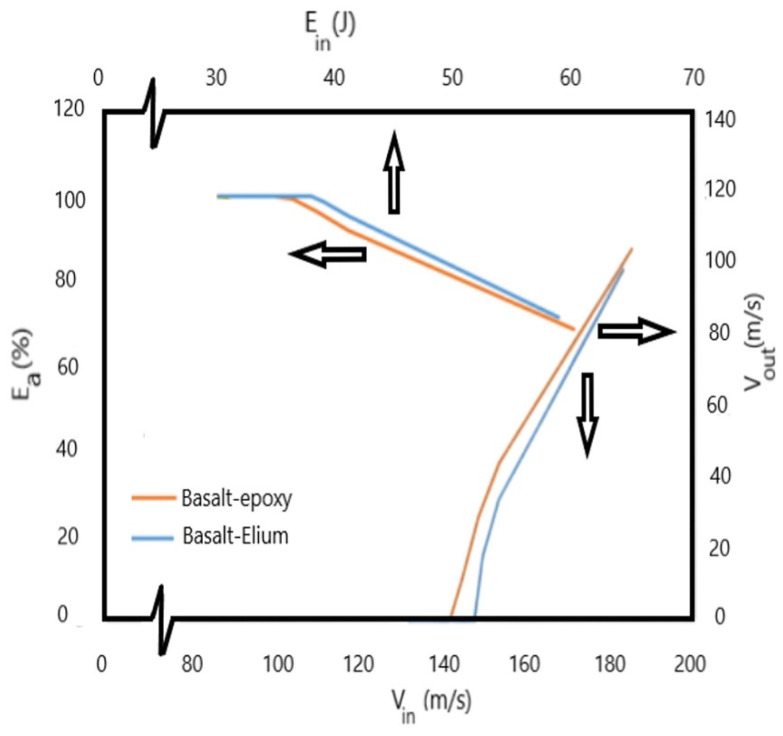
Graphs of impact velocity vs. velocity after penetration and energy incident vs. percent absorbed energy of the two composites.

The changes in the velocity of the projectile after HVI into the two composites for different projectile velocities are shown in Figure 11. The figure thus represents the ballistic limits of the composites. As can be seen from the results, the ballistic limit of the composite made with the Elium-based matrix is greater than that made with epoxy resin, which is attributed to the more ductile and higher fracture toughness of Elium compared to epoxy, which in turn facilitates the absorption of more energy when compared to a less ductile epoxy. This increased energy absorption is in proportion to the strain energy of the matrix. Elium has a larger strain energy than epoxy.

The variations observed in the graphs illustrated in Figure 11 indicate that the incident velocity increases while the energy absorption remains mainly constant but that they converge at extreme values. This convergence is due in part to the effectiveness of the resin in absorbing energy reduced with significantly higher velocities, while the fiber becomes the dominant loading constituent. 

## 5. Numerical Simulations

Finite element software LS-DYNA (ANSYS Inc., Livermore, CA, USA, 2023) was used in this study. The LVI and CAI models were created using LS-PREPOST V4.9 and evaluated on a workstation using LS-DYNA V13.1.

### 5.1. Models’ Configuration

**Figure 12:**
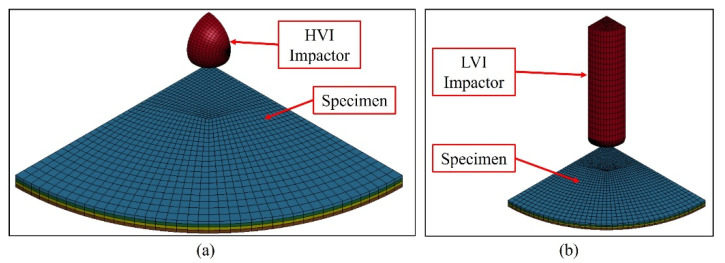
Quarter-symmetry finite element models: (**a**) HVI model; (**b**) LVI model.

Figure 12 shows an overview of the models developed to simulate the response of the specimens under HVI and LVI, respectively. Both models modelled the specimens with the same configurations; the only difference was the impactor’s geometry. Quarter symmetry was utilized to maximize computational efficiency. The HVI and LVI impactors were modelled to reflect the experimental counterparts and were initially placed only 0.1 mm away from the specimen to minimize computational cost. An initial velocity was applied to model the impacts.

The HVI and LVI impactors were represented in the model using solid elements (ELFORM=1) and characterized as rigid materials (MAT_20). Their movement was confined solely to the vertical (z-direction) in the material card. Adjustments were made to the LVI impactor’s material density to ensure that its mass matched that of the experimental counterpart, while the HVI impactor’s material properties were kept the same as those of steel. Eroding contact was defined between the impactor and a designated part set encompassing all specimen components. For contact stability, the pinball segment-based contact penalty formulation was adopted.

**Figure 13:**
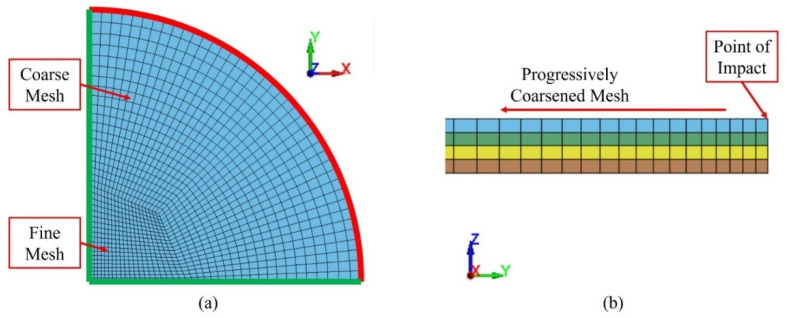
Details of specimen meshing: (**a**) top view; (**b**) cross-section view.

It was assumed that the specimen was fully restrained by the impact fixture; therefore, only the unsupported region of the specimen was modelled. More details of the specimen model are shown pictorially in Figure 13. The center region was modelled using a relatively fine mesh (0.5 mm element size), while the mesh was coarsened progressively towards the boundary. Symmetry boundary conditions were applied to the nodes along the green lines In Figure 13a, and the boundary (red arc) was fully restrained.

### 5.2. Element Choice and Mesh

Figure 13b shows a cross-section view of the specimen model. The specimen was modelled with four layers of tShell elements (ELFORM=1) in the thickness direction, representing the four layers of biaxial fabrics in each specimen. Delamination at the interfaces between neighboring tShell layers was modelled using the cohesive zone method.

The use of a single through-thickness integration point can potentially cause instability in the cohesive zone used to simulate interfacial contacts; thus, it was intentionally avoided. Since ELFORM=1 is not compatible with either two or four through-thickness integration points, three through-thickness integration points were defined for all Tshell elements. 

### 5.3. Material Models

The basalt–Elium layers were modelled using MAT_54 [48]. This material type effectively models an arbitrary orthotropic material in conjunction with the Chang–Chang failure criterion. It is important to highlight that the erosion of elements in MAT_54 does not rely solely on the failure criterion. Instead, four strain values are essential for determining the failure of a material: tension in the fiber direction (DFAILT), compression in the fiber direction (DFAILC), tension and compression in the matrix direction (DFAILM), and the in-plane shear (DFAILS). When failure is determined using the following criterion, the corresponding integration point gets deleted, and the stresses are set to zero. 

When the DFAILT parameter (or other parameters) is set as zero, the integration point is deemed failed only when the Chang–Chang failure criterion is satisfied in the tensile fiber mode.

If DFAILT is assigned a non-zero value, the integration point failure is determined by the strain surpassing any defined limits, including DFAILT, DFAILC, DFAILM, and DFAILS. In this case, the Chang–Chang failure criterion only determines the maximum stresses for all directions.

Subsequently, the element erodes when all through-thickness integration points fail. The mechanical properties of the FRP constituents are reported in Table 5.

Lastly, as mentioned earlier, the impactors are represented using a “rigid material model” (MAT_20). Although an elastic modulus of steel (205 GPa) is defined in the property card, these parts do not deform, and the elastic modulus is solely used for calculating contact stiffness.

### 5.4. Delamination Modelling

The plies interfaced, and the potential delamination and the subsequent delamination growth were modelled by incorporating a cohesive zoned modelling technique (CZM). Traditionally, CZM can be implemented by creating cohesive elements in the area of interest in LS-DYNA. Alternatively, CZM can be incorporated by modelling the interface through a surface-to-surface tiebreak contact algorithm. In this study, LS-DYNA’s CZM model (MAT_138), a bilinear mixed-mode relative displacement CZM model, was incorporated into the surface-to-surface tiebreak contact by selecting the value of the OPTION variable as 9. This option enforces a discrete crack according to the power law and Benzeggagh–Kenane (B-K) damage models. In comparison to the conventional cohesive element methods, this approach simplifies the modelling process. In this study, the bonding between 3D core and surface reinforcements was simulated using this technique.

**Figure 14:**
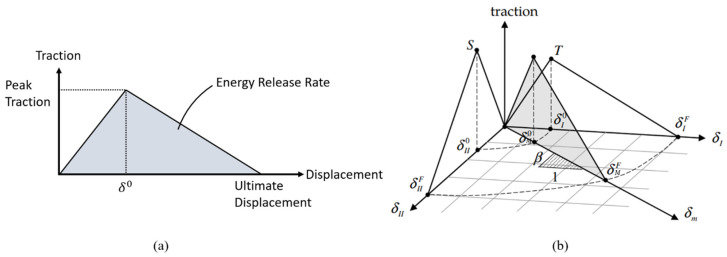
Bilinear CZM models: (**a**) the single-mode model; (**b**) the mixed-mode model.

This modelling methodology correlates bonding forces to relative displacements, encompassing normal and tangential directions corresponding to mode I and II fractures. Figure 14a illustrates the bilinear cohesive model in a single fracture mode. This model’s traction increases linearly concerning the relative displacement between the bonded parts. The initial slopes for mode I and mode II are expressed in units of stress/length. After the displacement reaches a critical value ($\delta^{0})$, the traction reduces linearly, simulating crack propagation and subsequent stiffness reduction. Ultimately, when the traction diminishes to zero at the ultimate displacement, it signifies complete interface debonding. Enclosed areas within the bilinear models symbolize energy release rates in the respective modes. Understandably, the most realistic debonding would be that caused by the combination of both modes. The mixed-mode bilinear model would consist of a proportion of each mode; this is illustrated graphically in Figure 14b, where β represents the proportionality. The mixed-mode critical displacement, $\delta_{M}^{0}$, and the ultimate displacement, $\delta_{M}^{F}$, are defined by Equations (4) and (5), respectively.

(4) $$\delta_{M}^{0}=\delta_{I}^{0}\delta_{II}^{0}\sqrt{\frac{1+\beta^{2}}{{{\left(\beta \delta_{I}^{0}\right)}^{2}+(\delta_{II}^{0})}^{2}}}$$

(5) $$\delta_{M}^{F}=\frac{2(1+\beta^{2})}{\delta_{M}^{0}}{\left[{\left(\frac{CN}{ERATEN}\right)}^{PARAM}+{\left(\frac{CN\times CT2CN\times \beta^{2}}{ERATES}\right)}^{PARAM}\right]}^{-\frac{1}{PARAM}}$$

The parameters used in the models were also adopted from [3] and are summarized in Table 6. 

### 5.5. Numerical Results

#### 5.5.1. LVI Results

**Figure 15:**
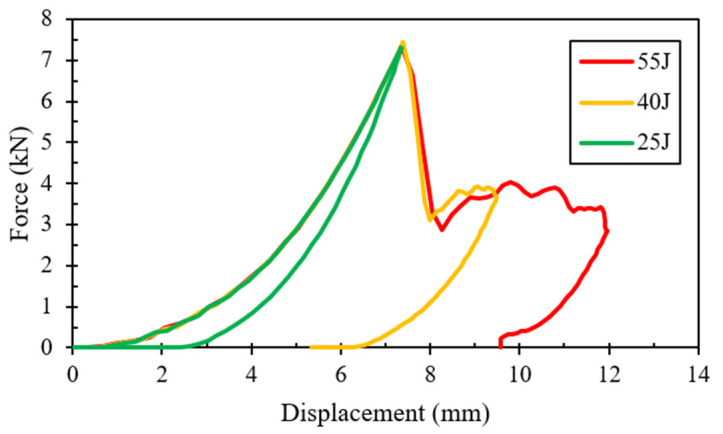
Numerically predicted LVI response.

The LVI response is shown in Figure 15. At 25 J, the response is smooth in both the indent and rebound process, indicating minimal damage to the specimen. The small area under the curve also confirms the minimal energy absorbed by the specimen. At 40 J, the distinct sudden drop after the peak load indicates significant damage to the specimen; however, the specimen rebounded against the impactor. At 55 J, even more energy was absorbed by the specimen. Despite the larger ultimate indentation, the specimen still successfully resisted the impactor. These observations are in reasonable agreement with the averages of the full experimental results illustrated in Figure 4. 

**Figure 16:**
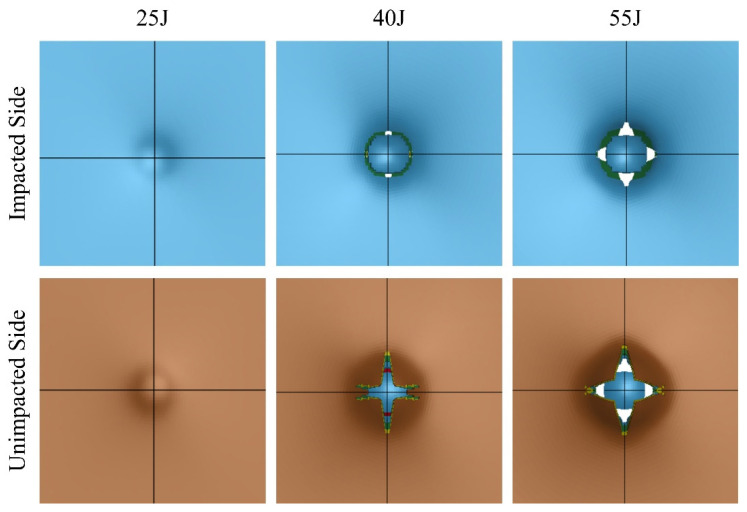
The numerically predicted low-velocity impact failure modes of basalt-Elium composites.

The numerically predicted damage modes are illustrated in Figure 16. The damage modes are compared with the representative experimentally observed modes shown in Figure 6, which evidences a close comparison.

#### 5.5.2. HVI Results

**Figure 17:**
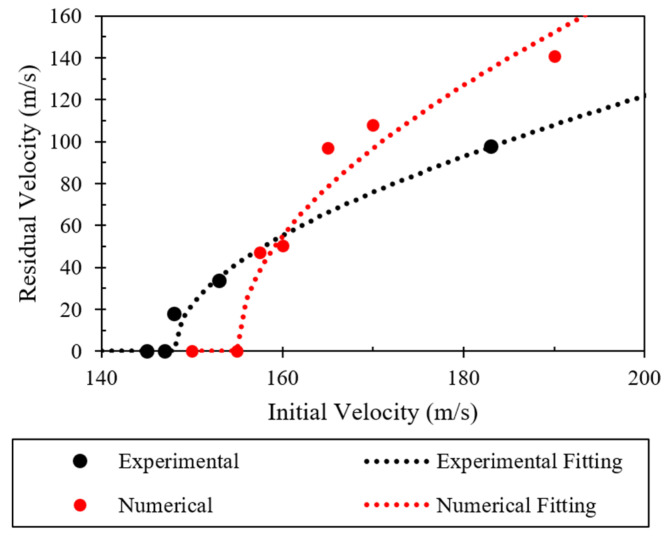
HVI numerical results fitted using Recht and Ipson’s model.

Figure 17 displays a comparison of the numerical and experimental results by plotting the residual velocity vs. the initial velocity. The data were fitted using Recht and Ipson’s model to obtain the curve [45]. The numerically determined Recht and Ipson’s parameters for the basalt–Elium composite are a=1.387 and p=2. The figure also presents the experimentally obtained data and curve. While one may initially perceive a large discrepancy between the numerical and experimental results, there is, however, a mere discrepancy of 5.7% between the predicted and experimental ballistic limits (157 m/s versus 148 m/s). The numerical model underestimated the energy absorption after penetration, possibly due to the microscale cracks in the composite and the existence of voids, which were not accounted for by the model; however, the aim of plotting post-penetration data points was to facilitate the more accurate evaluation of the ballistic limits, which, as mentioned earlier, is one of the most important parameters in an HVI study. The efficient simulation time and the prediction of ballistic limits with a reasonable accuracy promises the potential of the developed numerical model for future analysis.

**Figure 18:**
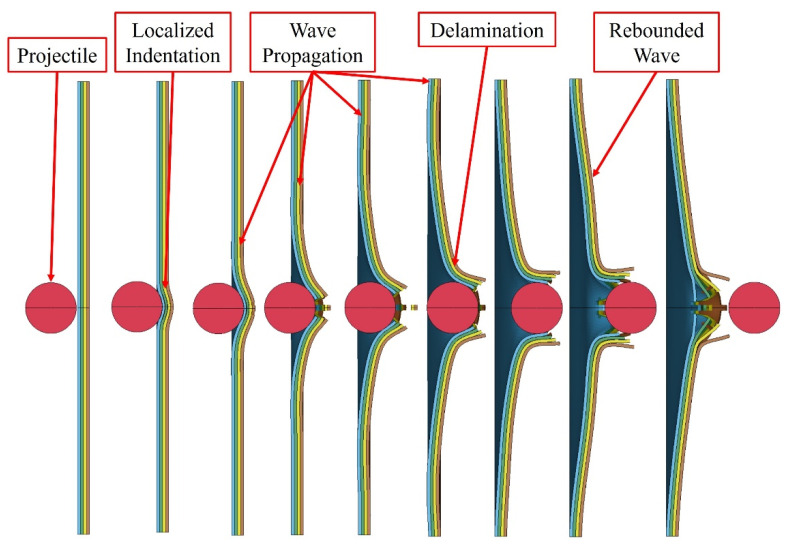
Numerically predicted HVI penetration process.

The numerically predicted penetration process is shown in Figure 18. As can be seen, the impact-induced wave propagated towards the boundary and rebounded back towards the center. Moreover, one can see that the failure in the composite first occurred on the unimpacted side. Interlaminar failure can also be observed throughout the process. The coloring of the constituents is consistent with the colors shown and identified in Figure 13.

The verified model can be effectively used to simulate the impact response of various composites and be used as a basis for optimizing the response of a given composite. 

## 6. Summary and Conclusions

The main goal of this research was to characterize the mechanical performance of a fully sustainable and recyclable composite, namely, basalt–Elium compared to its basalt–epoxy counterpart, particularly focusing on their responses to low- and high-velocity impacts (LVIs and HVIs, respectively). This study aimed to enhance the critically limited database for basalt–Elium composites. Basalt is an eco-friendly fiber combined with Elium, rendering a reformable, recyclable, and environmentally friendly composite material with remarkable mechanical properties, suitable for structural applications. The LVI impact tests were conducted with 25, 40, and 55 J impact energies. The HVI tests were conducted using an established method to evaluate the ballistic limits of the composites. Furthermore, effective and robust non-linear finite element models were developed and verified for simulating the LVI and HVI responses of the composites.

The following conclusions are drawn based on the results obtained from the investigation:

- Due to the low viscosity of Elium compared to epoxy, the fiber tows are wetted more thoroughly, which in turn increases the shear properties of its composites.
- Basalt–Elium exhibited a more effective LVI impact response compared to its epoxy-based counterpart. Basalt–Elium sustained an average force of 8.83 kN at an impact energy of 55 J, whereas the force taken by basalt–epoxy was 1.2 kN. Basalt–epoxy specimens exhibited more brittle failure modes with a higher degree of penetration during impact events than basalt–Elium specimens.
- The HVI test results demonstrated similar effectiveness for basalt–epoxy compared to basalt–Elium, with basalt–epoxy achieving the highest ballistic limit of 148 m/s versus 142.5 m/s for basalt–epoxy. Similar failure modes could be observed between the LVI and HVI responses, with basalt–Elium showing more apparent elastic–plastic behavior.

The numerical models developed in this study proved to be robust and fairly accurate in simulating the LVI and HVI responses of the composites. The models could accurately capture the failure mode compared to the experimentally observed modes. Moreover, one could clearly discern the damage modes and impact-induced wave propagations in the simulations. Moreover, the models can be used to investigate the responses of various components made of such composites, thereby eliminating the requirement for conducting time-consuming and costly experiments requiring specialized equipment.

## Figures and Tables

**Table 1:** Basic mechanical properties of basalt fiber and its composites.

Material	Tensile Strength (MPa)	Tensile Modulus (GPa)	Density (g/cm^3^)	Shear Strength (MPa)	Shear Modulus (GPa)	Void Content	% Fiber Weight
Basalt *	4840	89	2.70				
		13.854	1.59	36.8	2.667	6.54	54.3
Basalt–Elium	386.8	16.566	1.49	39.9	3.365	5.51	57.6

* The values for basalt fiber were extracted from [41]; all other values were evaluated in-house.

**Table 2:** Theoretical ballistic limit variable values of basalt.

Parameter	Thickness (mm)	$\sigma_{e}\;(MPa)$	ρ (kg/m^3^)	Γ	*D* (mm)	*M* (kg)
Values	2	164	1590	1.5	9.53	0.0035

**Table 3:** Average values of force for each low-velocity impact energy for the composites.

Material	Average Peak Force (N)	Impact Energy (J)	Standard Deviation	Coeff. of Variation
Basalt–epoxy	7165.22	55	617.11	18.02
	6886.80	40	244.49	10.49
	6029.54	25	88.67	1.48
Basalt–Elium	8831.26	55	518.70	5.87
	7942.06	40	718.62	9.05
	6484.40	25	511.77	7.89

N stands for Newtons and J for Joules.

**Table 4:** Ballistic limit velocity of the composites.

Basalt–Epoxy			Basalt–Elium		
$V_{l}\;(m/s)$	a	p	$V_{l}\;(m/s)$	a	p
143	0.920	2	148	0.907	2

**Table 5:** Values of the parameters used in MAT_54.

Properties *	Equivalent Generic Symbols	Basalt–Elium Crossply
$\mathrm{RO}\;\left(kg/m^{3}\right)$	ρ	1500
$\mathrm{EA}\;\left(Pa\right)$	E_11_	$1.708\times 10^{10}$
$\mathrm{EB}\;\left(Pa\right)$	E_22_	$1.708\times 10^{10}$
$\mathrm{EC}\;\left(Pa\right)$	E_33_	$8.93\times 10^{9}$
PRBA	ν_21_	0.055
PRCA	ν_31_	0.3265
PRCB	ν_32_	0.3265
$\mathrm{GAB}\;\left(Pa\right)$	G_12_	$3.366\times 10^{9}$
$\mathrm{GBC}\;\left(Pa\right)$	G_23_	$3.366\times 10^{9}$
$\mathrm{GCA}\;\left(Pa\right)$	G_31_	$3.366\times 10^{9}$
DFAILM	ε_22(T&C)_ **	0.1422
DFAILS	γ_12_	0.3
DFAILT	ε_11(T)_	0.1422
DFAILC	ε_22(C)_	−0.1422
$\mathrm{XC}\;\left(Pa\right)$	σ_11(C)_	$3.868\times 10^{8}$
$\mathrm{XT}\;\left(Pa\right)$	σ_11(T)_	$3.868\times 10^{8}$
YC (Pa)	σ_22(C)_	$3.868\times 10^{8}$
$\mathrm{YT}\;\left(Pa\right)$	σ_22(T)_	$3.868\times 10^{8}$
$\mathrm{SC}\;\left(Pa\right)$	τ_22_	$3.990\times 10^{7}$

* Designations as per the LS-DYNA User’s Manual [43]. ** T and C stand for tensile and compressive, respectively.

**Table 6:** CZM tiebreak contact parameters [49].

NFLS (Pa)	SFLS (Pa)	PARAM	$\mathrm{ERATEN}\;(J/m^{2})$	$\mathrm{ERATES}\;(J/m^{2})$	CT2CN	CN (Pa)
$5.90\times 10^{7}$	$2.30\times 10^{7}$	1	1500	2000	0.4286	$3.5\times 10^{12}$

## Data Availability

The data presented in this study are available on request from the corresponding author. The data are not publicly available due to restrictions under a non-disclosure agreement (NDA) with the material provider, which limits the sharing of specific data outside of pre-approved parties.
